# Structure‐informed engineering of plant–microbe interactions

**DOI:** 10.1111/tpj.70986

**Published:** 2026-07-03

**Authors:** Gloria Meng‐Hsuan Lin, Tim Lange, Alexander Förderer

**Affiliations:** ^1^ Max‐Planck‐Institute of Molecular Plant Physiology 14476 Potsdam‐Golm Germany

**Keywords:** immunity, root symbiosis, NLR, structure‐guided engineering, arbuscular mycorrhiza, root nodule symbiosis, receptors

## Abstract

This review critically evaluates how structural biology has enabled interface‐informed engineering of plant–microbe interactions, with a clear emphasis on the relative maturity of plant–pathogen research compared with symbiosis engineering. In plant immunity, atomic resolution structures of apoplastic receptors, host targets, and intracellular nucleotide‐binding leucine‐rich repeat receptors (NLRs) were already translated into concrete engineering strategies, including altered effector recognition, expansion of specificity, effector‐insensitive host variants, and mitigation of autoimmune phenotypes. These studies collectively demonstrate that structure‐guided approaches can move beyond descriptive insight to predictive and functional receptor design. Meanwhile, the rapidly accumulating structural information on symbiosis‐related receptors, signaling components, and nutrient‐sensing pathways indicates that engineering of symbiosis is an emerging new frontier. Structures of LysM receptors, symbiotic co‐receptors, calcium channels, transcriptional regulators, and hormone receptors reveal mechanistic parallels to immune signaling, including ligand discrimination, allosteric activation, and signal integration. The manuscript argues that symbiosis engineering can explicitly draw on conceptual and methodological templates established in pathogen resistance, such as interface remodeling, domain swapping, gain‐of‐function channel variants, and regulatory buffering to avoid deleterious outcomes. By juxtaposing these two fields, the review identifies transferable design principles and current limitations, and outlines how lessons from structure‐guided immunity engineering may accelerate rational manipulation of beneficial plant–microbe interactions for sustainable crop improvement.

## Significance Statement

Plant‐microbe interactions profoundly affect crop productivity through both beneficial symbioses and pathogenic infections. This review highlights how advances in protein structural biology have transformed our understanding of these interactions and are paving the way for structure‐guided engineering of plant‐microbe molecular interfaces.

## ENGINEERING OF IMMUNE RECEPTORS AGAINST PATHOGENS

Immunity against pathogens is mediated both through recognition of their presence in the apoplast and in the cytoplasm. Over the past years, many protein structures (Table 1) were determined and frequently exploited for structure‐guided engineering of plant–pathogen interactions that will be highlighted in the following.

**Table 1:** Structural studies of proteins involved in plant–pathogen interactions

Plants, pathogens	Protein(s)	PDB	References	Method	Comments
*A. thaliana*	FLS2‐flg22‐BAK1	4MN8	Sun et al. (2013)	X‐ray	Flg22 epitope
*N. benthamiana*, *P. sojae*	RXEG1‐XEG1	7W3V	Moreno Jimenez et al. (2024)	EM	
	RXEG1‐XEG1	7DRB		X‐ray	
	RXEG1‐XEG1‐BAK1	7DRC		EM	
*P. vulgaris*, *F. phyllophilum*	PGIP2‐PG	8IKW	Xiao et al. (2024)	X‐ray	
*A. thaliana*	ZAR1‐RKS1‐PBS2^UMP^	6J5T	Wang et al. (2019)	EM	Dicot CNL resistosome
*T. monococcum*, *P. graminis*	Sr35‐AvrSr35	7XC2	Förderer et al. (2022)	EM	Monocot CNL resistosome
*H. vulgare*, *B. graminis*	MLA‐Avr_A13_‐1	9FYC	Lawson et al. (2025)	EM	
*O. sativa*, *M. oryzae*	Pikh‐1 HMA‐AvrPikC	7A8X	de la Concepcion et al. (2021)	X‐ray	
	Pikp‐1 HMA‐AvrPikC	7A8W			Engineered
	Pikm‐1 HMA‐AvrPikE	6FUB	de la Concepcion et al. (2018)	X‐ray	
	Pikm‐1 HMA‐ AvrPikA	6FUD			
*O. sativa*, *P. oryzae*	Pikp‐1 HMA‐AvrPikC	7QPX	Maidment et al. (2023)	X‐ray	Engineered
	Pikp‐1 HMA‐AvrPikF	7QZD			Engineered
	HIPP43‐Pwl2	8R7A	Zdrzalek *et al*. (2024)	X‐ray	
O. sativa, P. oryzae	Exo70F2‐Avr‐Pii	7PP2	de la Concepcion et al. (2022)	X‐ray	
*A. thaliana*, *H. arabidopsidis*	RPP1‐ATR1	7CRB	Ma et al. (2020)	EM	LRR‐ID‐ATR1 complex
		7CRC			TNL resistosome
*N. benthamiana*, *X. euvesicatoria*	ROQ1‐XopQ	7JLU	Martin et al. (2020)	EM	TNL resistosome
		7JLX			TIR domains
		7JLV			Tetramer core
*A. thaliana*	EDS1‐PAD4	7XDD	Huang et al. (2022)	EM	
	EDS1‐PAD4	7XEY		X‐ray	pRib‐ADP
	EDS1‐SAG101	7XJP	Jia et al. (2022)	EM	ADPr‐ATP
	EDS1‐PAD4‐ADR1	8ZW9	Yu et al. (2024)	EM	
	EDS1‐SAG101‐NRG1A	8YL6	Xiao et al. (2025)	EM	
	EDS1‐SAG101‐NRG1C	8YL7			
	EDS1‐SAG101‐NRG1C	8YN0	Huang et al. (2025)	X‐ray	ADPr‐ATP
*O. sativa*	EDS1‐PAD4‐ADR1	8ZF0	Wu et al. (2024)	EM	pRib‐ADP
*N. benthamiana*	NRC2	8RFH	Selvaraj et al. (2024)	EM	Dimer
		9FP6	Madhuprakash et al. (2024)	EM	Hexamer
*S. lycopersicum*	NRC2	8XUO	Ma et al. (2024)	EM	Dimer
		8XUQ			Tetramer
		8XUV			Filament
*S. lycopersicum*, *G. rostochiensis*	NRC1‐SS15	8BV0	Contreras, Pai, et al. (2023)	X‐ray	NB‐ABC domain

The species names listed sequentially here are *Arabidopsis thaliana*, *Nicotiana benthamiana*, *Phytophtora sojae*, *Proteus vulgaris*, *Fusarium phyllophilum*, *Triticum monococcum*, *Puccinia graminis*, *Hordeum vulgare*, *Blumeria graminis*, *Oryza sativa*, *Magnaporthe oryzae*, *Pyricularia oryzae*, *Hyaloperonospora arabidopsidis*, *Xanthomonas euvesicatoria*, *Solanum lycopersicum*, and *Globodera rostochiensis*. Methodologies to determine the structures listed here are X‐ray (X‐ray crystallography) and EM (cryogenic electronic microscopy).

Apoplastic immunity composes the first layer of plant immunity and is often triggered by concerted action of soluble apoplastic proteins and by pattern recognition receptors (PRRs), such as receptor‐like kinases (RLKs) and receptor‐like proteins (RLPs) residing in the plasma membrane (Schellenberger et al., 2019). Structure‐guided engineering and AlphaFold comparison of natural FLS2 alleles from *Glycine max* (*G. max*), *Vitis vinifera*, *V. riparia*, and *Quercus variabilis*, heavily based on the structure of the FLS2‐flg22‐BAK1 complex (PDB 4MN8) (Sun et al., 2013), enabled expansion and alteration of flagellin recognition (Li et al., 2025; Zhang, Liu, et al., 2025). In *Nicotiana benthamiana* (*N. benthamiana*), the apoplastic xylanase effector XEG1 from *Phytophthora sojae* is recognized by the leucine‐rich repeat domain (LRR) of the RLP RXEG1, inducing immunogenic cell death (Sun et al., 2022). Structural analyses of the XEG1‐RXEG1^LRR^ complex and the trimeric complex containing BAK1 (PDB 7W3V, 7DRB‐C) revealed that two loop‐out regions of the rigid RXEG1^LRR^ scaffold insert into β‐sheets at the XEG1 active site, mediating enzymatic inhibition and immune recognition (Sun et al., 2022). This example shows how nature tends to evolve pathogen recognition against functionally conserved effectors, a design principle that likely yields more durable resistance also in synthetic approaches.

Host apoplastic enzymes such as proteases and chitinases are key antimicrobial factors, but frequently targeted by pathogen‐derived small secreted proteins (SSPs). AlphaFold Multimer has emerged as a high‐throughput approach to predict cross‐species SSP‐hydrolase interactions and their inhibition modes, as validated using known complexes between tomato P69B/Pip1 and *Phytophthora* SSPs. This large‐scale screening identified 376 putative complexes, including 15 with previously undescribed inhibition types, several of which converged on the P69B subtilase, suggesting it functions as an effector hub. Overall, this interactome screening enables *in silico* discovery of plant–microbe interactions that are difficult to capture experimentally and can reveal host targets of pathogen effectors. Such insights not only aid effector annotation and hub identification but also provide a structural basis for engineering effector‐insensitive host proteins (Homma et al., 2023).

Another example, the plant polygalacturonase‐inhibiting protein (PGIP) from *Phaseolus vulgaris* (PGIP2) and a *Fusarium phyllophilum‐derived* polygalacturonase (PG) highlights how apoplastic LRR‐only proteins function in pathogen‐induced susceptibility. PGIP2‐PG structure (PDB 8IKW) showed two interfaces at the concave site of the PGIP2 N‐terminal LRR superhelix involving contacts between the PGIP2 LRR blades and the β‐sheets adopted by PG. By this, the LRR covers the PG like a lid, which does not lead to a complete inhibition of the PG, but to an altered catalytic activity resulting in the production of long‐chained oligogalacturonides conferring new resistance against *Phytophtora* in soybean seedlings (Xiao et al., 2024).

Therefore, *in silico* interaction screens between putative pathogen effectors and potential host targets could be used to: (I) Identify existing pairs of effector proteins and host virulence factors; and (II) Integrate structural mechanisms to manipulate the plant–microbe interface to promote the plant's immune signaling besides the qualitative resistance typically conferred by NLRs. Latter was chosen to convert the *Medicago truncatula* (*M. truncatula*) PGIP1 (PGIP1) into a PG‐binding protein by mimicking the PGIP2 interface at the corresponding PGIP1 surface using only four amino acid substitutions (PGIP1^4mut^) (Xiao et al., 2024). Moreover, further substitutions in the positively charged polygalacturonide binding site of PGIP1, namely, N274R and S276R, shifted the product profile of the formed PGIP1‐PG complex toward the production of long‐chained immunoactive oligogalacturonides (Xiao et al., 2024).

## ENGINEERING OF INTRACELLULAR RECEPTORS OF CEREALS

Intracellular nucleotide‐binding leucine‐rich repeat receptors (NLRs) sense pathogen effectors to induce a programmed cell death called hypersensitive response (HR) typically conferring a qualitative resistance in crops (Gu et al., 2025). NLRs act in different modes to initiate the downstream signaling events to establish such effector‐triggered immunity (ETI) (Islam et al., 2024). Engineering of effector recognition by NLRs has become a widespread strategy in plant research. In a landmark paper, Wang and colleagues described the first structure of the full‐length NLR HopZ‐activated Resistance 1 (ZAR1) from *Arabidopsis thaliana* (*A. thaliana*), a dicot, opening a new perspective of structure‐informed engineering of plant resistance (Wang et al., 2019). The structure revealed a pentameric resistosome composed of five ZAR1 protomers in an activated state with the resistance‐related kinase 1 (RKS1) and the uridylated protein kinase PBL2^UMP^ (PDB 6J5T) resembling a wheel‐like structure comparable with the previously reported structure of inflammasomes (Wang et al., 2019). As implied by the hydrophobicity of the N‐terminus and the central hole of this structure, electrophysiological experiments showed that the ZAR1 resistosome forms a calcium pore in the plasma membrane to initiate cell death (Bi et al., 2021).

In cereals, the pentameric structure of the activated wheat Sr35 in complex with the *Puccinia graminis* (*P. graminis*) effector AvrSr35 underlined the accepting action of the canonical singleton CNLs (PDB 7XC2) (Förderer et al., 2022; Zhao et al., 2022). Comparable to the activation mechanisms observed in the ZAR1 resistosome, Sr35 is activated by a steric clash of the AvrSr35 with the Sr35 nucleotide‐binding domain upon interaction, finally inducing its pentamerization to form a calcium‐permeable ion channel. Two Sr35 homologs from *Triticum aestivum* (*T. aestivum*) and *Hordeum vulgare* (*H. vulgare*) were engineered at the Sr35‐AvrSr35 interface, yielding a new recognition specificity of both gain‐of‐functions (GOF) toward the AvrSr35 effector (Förderer et al., 2022). Due to the remarkable conservation of the resistosome structure, allelic diversity among homologs can even be utilized for receptor engineering. Using Shannon entropy analyses, surface‐exposed residues particularly in the C‐terminal LRRs can be identified between the allelic wheat resistance proteins Sr50 and Sr33, swapping from which can transfer effector recognition specificity (Tamborski et al., 2023). These demonstrated how atomic structure of a cereal NLR can be harnessed for resistance engineering of naturally occurring alleles.

High level of structural conservation also allowed recombination of resistance of *H. vulgare Mla13/7* variants *in vitro* by generating a library of these two resistance genes, *Mla13* and *Mla7*. The obtained variants acquired novel binding specificity for the effector Avr_A13_‐V2, previously unrecognized by either template (Zhang et al., 2025). This highlights that gene shuffling among related resistance genes with detailed insights on the residues determining effector recognition is possible even when effectors are entirely unknown.

Another engineering example (Lawson et al., 2025) is based on the structure of barley powdery mildew resistance MLA13 complex effector Avr_A13_‐1 (PDB 9FYC), which adopted a well‐characterized RNAse‐like fold as related effectors from the same family (Cao et al., 2023). Notably, Lawson and colleagues identified the same polymorphism in the LRR‐Avr_A13_‐1 interface as previously studied MLA13 and MLA7, namely, the MLA7^L902^ and MLA13^S902^. Given the possibility that MLA13 may lack a canonical resistosome, future works will aim to either find a corresponding complex or elucidate the MLA13 activation mechanism.

Besides singleton NLRs, paired NLRs consist of two genetically linked NLRs encoding one sensor NLR and one executor NLR, enabling functional specialization toward sensing and signaling (Contreras et al., 2023). In *Oryza sativa* (*O. sativa*), several such NLR pairs are well characterized including the Pik‐1/Pik‐2, the Pii‐2 and Pii‐1, and the RGA5/RGA4, where the one NLR acts as cell death executor and the other as a pathogen sensing, allosteric activator. These sensor NLRs perceive a group of avirulence proteins from the fungal pathogen *Magnaporthe oryzae* (*M. oryzae*) named MAX effectors. *O. sativa* sensor NLRs share integrated domains (ID) as a common feature. In Pik‐1 and RGA5, the ID is a heavy metal‐associated (HMA) and mediates recognition of AvrPik effectors, whereas effector recognition of AvrPii by the Pii‐2 sensor is enabled by a nitrate‐induced (NOI) domain (Fujisaki et al., 2024).

The NLR Pik‐1 functions as an allelic series with distinct effector specificities: Pikp‐1 recognizes AvrPikD and AvrPikE, whereas Pikm‐1 and Pikh‐1 additionally recognize AvrPikA. In contrast, AvrPikC and AvrPikF evade resistance, failing to interact with full‐length Pik‐1 receptors or to elicit a HR *in planta*. These resistance‐breaking interactions have motivated structure‐guided studies of effector perception by the integrated HMA domain of Pik‐1 to enable rational receptor engineering.

Although AvrPikC and AvrPikF are not recognized *in planta*, both effectors retain binding to isolated Pik‐1 HMA domains. The structure of the AvrPikC‐Pikh‐1 HMA complex (PDB 7A8X) revealed that a single effector polymorphism (D67, corresponding to A67 in AvrPikE; PDB 6FUB) disrupts receptor recognition by reconfiguring the interaction interface. D67 forms an intramolecular bond with R64, preventing productive interaction with Pikh‐1 D244 (de la Concepcion et al., 2018, 2021), illustrating how subtle sequence variation overcomes binding and breaks immune activation.

The discovery that AvrPik effectors target heavy metal‐associated plant proteins (HPPs/HIPPs) fundamentally reshaped models of Pik‐1‐mediated recognition (Maidment et al., 2021). All AvrPik alleles, including the resistance‐breaking AvrPikC and AvrPikF, bind the *O. sativa* HIPP19 HMA domain with nanomolar affinity, even though these effectors evade detection by native Pik‐1 receptors. Structural comparisons between AvrPik‐HIPP19 and AvrPik‐Pik‐1 HMA complexes, including Pikm‐1‐AvrPikA (PDB 6FUD), revealed conserved binding architectures but a substantially expanded interface in the host–target complex (de la Concepcion et al., 2018; Maidment et al., 2021). Importantly, resistance‐breaking polymorphisms such as AvrPikF K78 lie within the interface yet do not impair HIPP19 binding, suggesting that increased interface complexity buffers sequence variation that disrupts Pik‐1 recognition. These findings provided both a mechanistic explanation for effector evasion and a blueprint for engineering receptors with expanded specificity.

Leveraging this insight, structure‐guided design was used to remodel the Pikp‐1 HMA domain toward AvrPikC and AvrPikF recognition (Maidment et al., 2023). Introduction of a single HIPP19‐inspired substitution (S258E) into wild‐type Pikp‐1 was insufficient for recognition. However, when incorporated into the previously broadened Pikp‐1^NK‐KE^ background, the resulting Pikp‐1^SNK‐EKE^ variant gained specific recognition of AvrPikC and AvrPikF while retaining AvrPikD perception. Structural analyses (PDB 7QPX, 7QZD) showed that E258 forms an additional stabilizing hydrogen bond at the interface, mirroring HIPP19 E72 and illustrating how host–target features can be selectively transferred to overcome effector polymorphisms (de la Concepcion et al., 2021; Maidment et al., 2023).

A complementary approach directly replaced the Pikp‐1 HMA with the HIPP19 HMA domain. Although the initial Pikp‐1^HIPP19^ chimera exhibited effector‐independent autoactivity, restoration of the degenerated metal‐binding loop eliminated this defect. The optimized Pikp‐1^HIPP19mbl7^ receptor conferred AvrPik‐dependent HR in *N. benthamiana* and resistance in transgenic *O. sativa* against strains carrying AvrPikC and AvrPikF (Maidment et al., 2023). This provided compelling functional evidence that Pik‐1 HMA domains act as host–target and that direct integration of effector targets can bypass evolutionary constraints on natural receptors.

**Figure 1:**
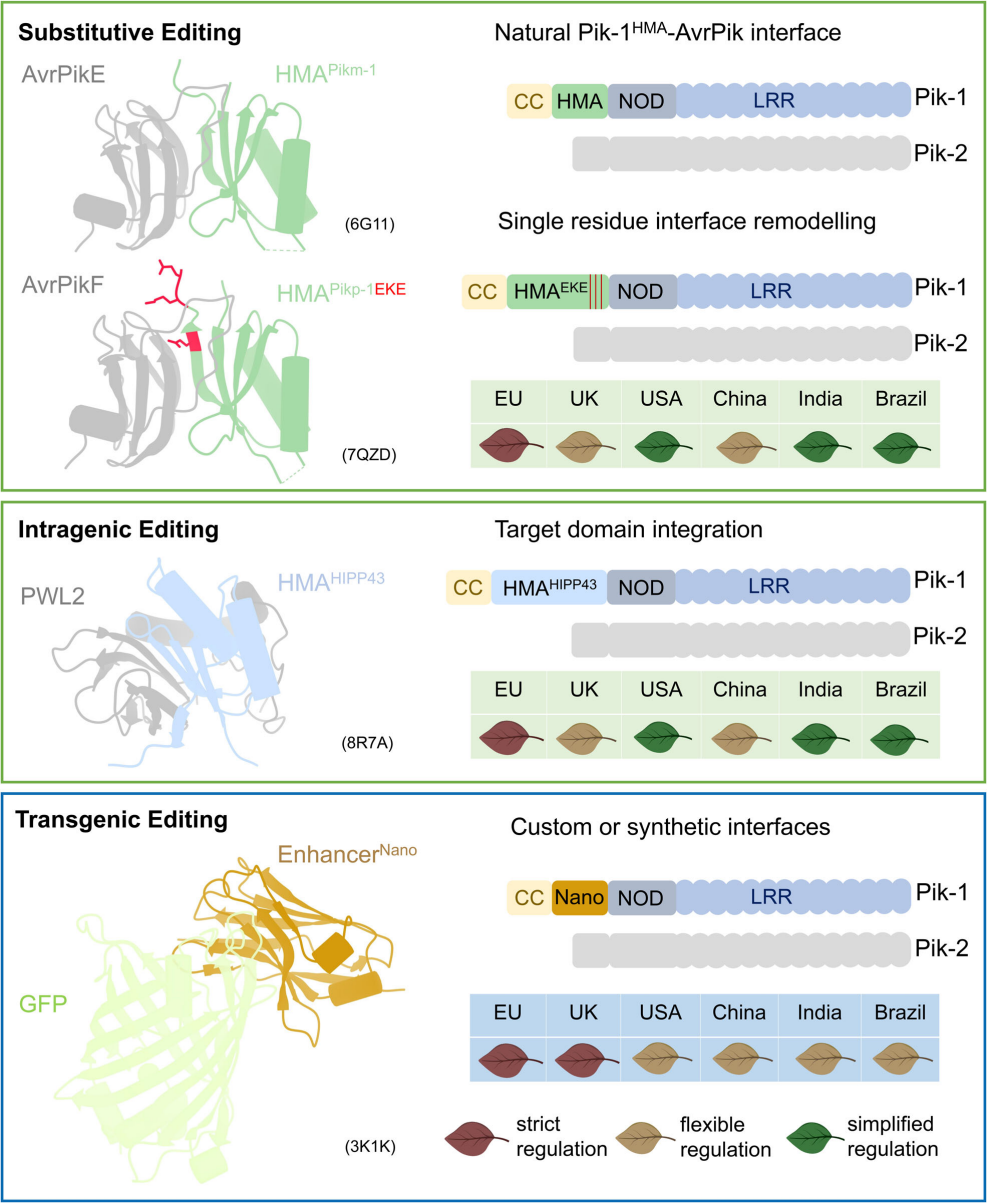
Regulation of genetically modified crops using NGT's (green outline) and transgenic (blue outline) approaches differs regionally according to the country's legislation. NGT's enable specific substitutive remodeling of natural interfaces. The exchange of homologous domains within one species, is often considered as intragenic editing often following simplified regulations, as the genetic material originates from a shared genetic origin and no transgenes were introduced. The exchange of genetic material across species is often regulated more strictly in the EU and the UK. Represented protein structures were taken from the RCSB Protein Data Bank (PDBs 7QZD, 8R7A, 3K1K) and visualized using ChimeraX Version 1.11.1.

The optimization of effector recognition based on single amino acid substitution of native *R*‐genes (e.g., Pikp‐1^SNK‐EKE^) can be achieved by precise new genomic techniques (NGT's) and follows simplified or case‐by‐case regulation in the USA, China, India and Brazil, making this a feasible approach for resistance engineering in crops species (Figure 1). Similarly, the generation of genetic chimera within one species (e.g., the integration of HIPP HMA into Pik‐1 NLR in *O. sativa*), does not introduce foreign genetic material and is therefore often legalized following simpler regulation than transgenic species (Figure 1).

Related studies on the RGA5/RGA4 system underscore both the power and the limits of structure‐guided ID engineering. While engineered RGA5 HMA variants acquired AvrPikD binding and triggered cell death in heterologous systems, resistance in *O. sativa* required additional modifications to restore executor compatibility (Cesari et al., 2022; Maqbool et al., 2015; Zhang et al., 2024). Together, these studies reveal a unifying principle: expanding effector binding is necessary but not always sufficient, for successful resistance engineering must integrate effector affinity with preserved NLR regulatory function.

Cereal NLRs have also enabled an innovative engineering strategy in which adaptive antibody‐like recognition is fused to plant immune receptors to create “made‐to‐order” resistance. In this approach, the integrated HMA domain of the sensor NLR Pikm‐1 was replaced with single‐domain antibody fragments (nanobodies), generating so‐called Pikobodies that specifically recognize non‐native targets such as GFP or mCherry. Several Pikobody variants triggered a specific HR only in the presence of their cognate fluorescent protein and, when stably expressed in *N. benthamiana*, conferred resistance to PVX‐GFP comparable to natural Rx‐mediated immunity, demonstrating the feasibility of synthetic effector recognition (Kourelis et al., 2023).

Beyond nanobody fusions, recent advances in machine learning‐based protein design (e.g., RFdiffusion) offer powerful routes to generate bespoke protein binders with defined interfaces, symmetries, and functions (Watson et al., 2023). Such computationally designed binders could substitute integrated domains in sensor NLRs, potentially enabling highly diverse and tunable recognition specificities. Importantly, future designs may incorporate regulatory features that control downstream NLR activation, helping to avoid autoimmunity while preserving robust immune outputs.

Besides the advantage of custom resistances, the integration of mammalian nanobodies or synthetically designed binders is likely considered a transgenic approach and is still strictly regulated for cultivation and human consumption (Figure 1).

A recent preprint showed a random mutagenesis approach to create a variety of gain‐of‐function or loss‐of‐function receptor variants which need to be screened efficiently to select toward an increased affinity against a certain effector. Via the Pik‐1/AvrPik system, directed evolution using fluorescence activated cell sorting (FACS) provides an efficient pipeline to screen for new binding affinities among Pik‐1 variants. A library of approximately 2.2 × 10^7^ Pikh‐1 HMA variants was then created by error‐prone PCR, which were screened for AvrPikC, AvrPikF, and AvrPiz‐t interaction. Interestingly, polymorphisms providing a stable recognition were not limited to the three described interfaces known from HMA/AvrPik structures (Toghani et al., 2025).

The identification of NLR pairs is traditionally based on genetic linkage analysis and rational approaches like the presence of an ID in the putative sensor partner (Cesari et al., 2022; Sang et al., 2025). Recent inspection of structural predictions to differentiate sensor and executor NLRs in pairs was reported. Using AlphaFold3 prediction, modeling of putative executor N‐terminal parts yielded higher predicted template modeling (pTM) scores than the putative sensor NLR. Moreover, the presence of the funnel‐like structure in the final model built by the α1 helix of the coiled‐coil domain indicates a direct membrane association and ion channel activity for the identified executor, as observed for the singleton NLRs ZAR1 and Sr35. Besides the successful grouping of the famous NLR pairs Pikm‐1/Pikm‐2, RGH2/RGH3, Pia‐2/Pia‐1, and Pii‐2/Pii‐1, a total of 10 previously proposed but further uncharacterized NLR pairs were analyzed, yielding a successful identification of a sensor and executor per pair, based on their pTM score and coiled‐coil structure. Hence, AlphaFold3 predictions can give a good indication to identify sensor and executor NLR when a given putative pair is already suggested. For this circumstance, NLRs were considered when they were identified in head‐to‐head clusters, belong to different phylogenetic clades, and consist of a complete N‐terminal domain (Toghani et al., 2025).

Engineering of NLR is frequently reported to cause autoimmunity. Consequently, autoactivity presents a bottleneck for the design of synthetic NLR (Bentham et al., 2023; Kourelis et al., 2023; Tamborski et al., 2023). To overcome the autoactivity observed in the engineered Pikp‐1^HIPP19^, P‐loop mutations in Pikp‐2 MHD motif (Pikp‐ 2^K217R^) were introduced, resulting in a reduced autoactivity of the Pikp‐1^HIPP19^/Pikp‐2 pair. Complete loss of autoactivity with a robust recognition of the AvrPikC and AvrPikF effectors was established by mutating seven amino acids of the metal‐binding motif in the β1‐α1 loop back to the residues found in native Pikp‐1 to generate the Pikp‐1^HIPP19mbl7^ (Maidment et al., 2023). Comparably, the introduction of nanobodies into Pikm‐1 for GFP and mCherry recognition resulted in autoimmune activity in five out of 11 tested variants using the Pikm‐2 executor and the replacement of the Pikm‐1 HMA by the RGA5 HMA in the Pikm‐1^RGA5^/Pikm‐2 resulted in autoimmunity in transient assays (Bentham et al., 2023; Kourelis et al., 2023). These challenges underline that engineering approaches of NLRs and their IDs need to consider the regulatory context of NLRs acting in pairs or networks and the regulatory relationships between Pik‐1 sensor alleles and Pik‐2 executor alleles provides a useful tool to avoid autoimmune phenotypes (Bentham et al., 2023).

Transient expression assays in *N. benthamiana* revealed that mismatching Pikp‐1 with the Pikm‐2 executor is sufficient to induce constitutive, effector‐independent cell death due to a single polymorphism in the Pikm‐2 NOD module (E230), highlighting extreme sensor‐executor co‐adaptation within the Pik NLR pair. This incompatibility is sensor‐dependent, as Pikm‐1 can be paired with either Pikm‐2 or Pikp‐2 without autoactivity, suggesting that the Pikm‐1 HMA domain has co‐evolved to buffer the autoactive potential of Pikm‐2, a function that cannot be substituted by Pikp‐1 (Bentham et al., 2023). Consequently, engineering of Pik‐1 receptors or HMA exchanges is particularly prone to autoimmune phenotypes when combined with Pikm‐2.

Exploiting this incompatibility, allele mismatching can also suppress autoactivity. Pairing Pikm‐1‐based engineered receptors with Pikp‐2 prevents constitutive activation while retaining effector‐dependent responses, as shown for Pikm‐1‐RGA5 chimeras and nanobody‐fused Pikm‐1 variants (Bentham et al., 2023, Kourelis et al., 2023). This strategy was also used to stabilize the engineered Pikm‐1^HIPP43^ receptor (Zdrzałek et al., 2024).

Beyond MAX effectors, AvrPii defines a distinct recognition paradigm. AvrPii binds exocyst components Exo70F2/3 and adopts a zinc‐finger fold (PDB 7PP2) unrelated to MAX effectors (de la Concepcion et al., 2022). Two preprints further focus on the role of exocyst components in plant immunity, showing that Exo70F2/3 interact with the NOI motif of the Pii‐2 sensor, and AvrPii disrupts this complex to trigger immunity (Fujisaki et al., 2024). Notably, integrated Exo70 domains occur in numerous NLRs, including the *H. vulgare* RGH2/RGH3. Structure‐guided engineering of the RGH2 Exo70 domain enhanced AvrPii affinity without autoactivity and conferred resistance in transgenic *H. vulgare*, demonstrating that exocyst‐based IDs provide a versatile platform for rational NLR engineering (Saado et al., 2024).

## ENGINEERING OF INTRACELLULAR RECEPTORS OF DICOT CROPS

In dicots, TIR‐NLRs represent an alternative signaling route toward immunogenic HR that is absent or rudimentary in monocots (Lapin et al., 2022). TIR‐NLRs (TNLs), such as Resistance to *Peronospora parasitica* 1 (RPP1) from *A. thaliana* and Recognition of XopQ 1 (ROQ1) from *N. benthamiana*, also form resistosomes. These TIR‐resistosomes display a tetrameric, clover‐leaf shaped structure (RPP1 PDBs 7CRB‐C; ROQ1 PDBs 7JLU‐V, 7JLX) (Ma et al., 2020; Martin et al., 2020). Lately, two preprints determined similar TNL resistosome structures of the *Linum usitatissimum* M and the *A. thaliana* WRR4A (Maruta et al., 2026; Zhao et al., 2026). To induce downstream signaling, the N‐terminal TIR domains of the resistosome oligomerize asymmetrically building a dimeric TIR‐dimer to form two enzymatically active centers with NADase activity. The effector recognition of RPP1, ROQ1, and WRR4A is mediated by an interface at the concave LRR and a C‐terminal jelly‐roll ID (C‐JID), whereas effector recognition by M is based on LRR and NOD contacts as it lacks a C‐JID. These insights guided engineering of WRR4A^CCG47^, a receptor recognizing the avirulent CCG47 effector based on three amino acid substitutions only.

Primary cryo‐EM data show that M and WRR4A rest in a monomeric state in which the NOD module packs against the concave LRR, supporting the hypothesis that these TNLs follow a comparable steric clash activation like the CNL ZAR1. However, a distinct activation mechanism of the NLR's TIR domain remains speculative, as both resting monomers lack the TIR density.

The immune signaling of TIR‐only proteins and TNLs relies on the activation of the heterocomplexes formed between the lipase like proteins Enhanced Disease Susceptibility 1 (EDS1) and Phytoalexin Deficient 4 (PAD4) and Senescence‐Associated Gene 101 (SAG101) which in turn activate the helper NLRs of the Activated Disease Resistance 1 (ADR1) and N Requirement Gene 1 (NRG1) families. Selective interactions were shown, as the activated EDS1‐PAD4 complex interacts with the helper ADR1L, whereas the activated EDS1‐SAG101 complex exclusively interacts with NRG1A (Huang et al., 2022). By isolating the activated EDS1‐hetrocomplexes, the structures of the *A. thaliana* EDS1‐PAD4 (PDB 7XEY, 7XDD) and EDS1‐SAG101 (PDB 7XJP) enabled identification of different TIR‐produced signaling molecules captured by the EDS1 heterodimers (Huang et al., 2022; Jia et al., 2022). In contrast to the apo‐complexes of EDS1‐PAD4 and EDS1‐SAG101, the dimerizing C‐terminal EP domains of EDS1 and its respective partner enclose an ADPr‐ATP (ADP‐ribosylated ATP) or di‐ADPR in EDS1‐SAG101 and an pRib‐ADP (phosphoribosyl ADP) in the EDS1‐PAD4 complex. Comparable for both complexes, binding of the ADPr‐ATP or pRib‐ADP activates the EDS1‐PAD4/SAG101 complex resulting in allosteric changes in the EP domains of PAD4 and SAG101 forcing it to rotate anticlockwise away from EDS1 forming the binding pocket to almost bury the entire TIR‐produced molecule inside the ED interface (Huang et al., 2022, Jia et al., 2022). Structures of the *A. thaliana* (PDB 8ZW9) and *O. sativa* (PDB 8ZF0) EDS1‐PAD4‐ADR1^WHD‐LRR^ trimeric complex revealed that the pRib‐ADP‐induced conformational change of the PAD4^EP^ creates the corresponding interface to accommodate the ADR1^WHD‐LRR^, underlining that TIR‐signaling based on the EDS1‐PAD4‐ADR1 module is conserved between monocots and dicots (Wu et al., 2024; Yu et al., 2024). Comparable principles of activation were recently found for the *A. thaliana* EDS1‐SAG101‐NRG1A^WHD‐LRR^ complex (PDB 8YL7), finally inducing NRG1A oligomerization (Xiao et al., 2025). A conserved interface was shown for the interaction of the TIR‐activated EDS1‐SAG101 complexed with NRG1C (PDB 8YN0), providing a regulatory layer to counteract the NRG1A‐dependent immunity response by sequestering the activated EDS1‐SAG101 heterocomplex (Huang et al., 2025). However, complete resistosome structures of ADR1 and NRG1 helper families are unresolved to date. Nucleotide‐derived second messengers are increasingly recognized as key components of innate immune signaling, especially in plants. Despite this, their potential application via externally applied agrochemicals has yet to be systematically investigated.

Solanaceous plants as major crops containing species such as *Solanum lycopersicum* (*S. lycopersicum*), *Solanum tuberosum* (*S. tuberosum*), *Capsicum annuum* (*C. annuum*) and others have received attention in studying resistance mechanisms. In solanaceous plants, a specialized helper NLR network exists around the NLRs Required for Cell Death (NRC) type NLRs, which act in cooperation with the sensors Rx, Sw‐5b, and Bs2 (Wu et al., 2017). Interestingly, *N. benthamiana* NRC2 and NRC4 were found to form homo‐oligomeric complexes under native conditions (Liu et al., 2024; Selvaraj et al., 2024). NRC2 was found to rest in a homodimer state when purified alone from leaves, whereas incubation of the NRC2 dimer with the Rx NBD and ATP induces the previously reported NRC2 hexameric resistosome (Madhuprakash et al., 2024; Selvaraj et al., 2024). The NRC2 dimer structure shows two inter‐protomer contacts between the three‐helix bundle of the nucleotide‐binding domain and the adjacent LRR, and one interface including stacking of both N‐terminal LRRs against each other (PDB 8RFH) (Selvaraj et al., 2024). Comparable insight were obtained by Ma et al., revealing the *S. lycopersicum* NRC2 not only in a resting dimer, but also in an inactive homo‐tetramer and filament (PDB 8XUO, 8XUQ, 8XUV) (Ma et al., 2024). Similar to the NRC2 dimer from *N. benthamiana*, the *S. lycopersicum* NRC2 dimer is built by head‐to‐head arrangement. In both resting dimers, an ADP molecule is bound to the respective nucleotide‐binding domain, however, all inactive tomato NCR2 complexes contain inositol phosphate in a positively charged pocked formed between the LRR and the winged helical domain. The presence of inositol phosphate in an NLR, points at a potential connection between NLRs and phosphate metabolism and could provide an opportunity to interfere with the hardwired trade‐off in plants between growth and defense. It is hypothesized that assembly of dimers and tetramers of S. *lycopersicum* NRC2 is the basis for the formation of filamentous NRC2 polymers, enabling different levels of inhibitory self‐sequestration. While reinforced inhibition can increase the activation threshold of NLRs, defined amino acid substitutions in *N. benthamiana* NRC2 were shown to decrease this threshold (Selvaraj et al., 2024), thereby providing a framework to engineer tunable NLR activation dynamics. Interfering with the plant's immunity, a nematode effector inhibits the NRC activation, which will be discussed in a later section.

## ENGINEERING OF HOST TARGETS

Pathogen effectors target plant proteins to promote pathogens' fitness (Deb et al., 2017). However, annotation of effector proteins is quiet challenging, as many effector proteins do not exhibit sequence homology between different species and even withing one species as they are thought to have evolved by several duplication events and further divergence (Seong & Krasileva, 2023). Besides their diminished homology, several examples are known of effector families sharing a conserved fold, including the previously mentioned MAX effectors of *M. oryzae* and RNAse‐like effectors in *Blumeria graminis* (*B. graminis*) (Cao et al., 2023; Oliveira‐Garcia et al., 2024). Hence, recent attempts were made to annotate and characterize sequence‐unrelated but structural similar effectors (SUSSs) using AlphaFold2 predictions of more than 26.000 pathogen secretome proteins of 15 pathogenic fungi including biotrophic, hemibiotrophic, and necrotrophic species. Further clustering using sequence and structure data was performed for successfully modeled secreted proteins revealing that the vast majority of the identified homologs or analogous proteins. On the other hand, greatly expanded species‐specific effector families were identified including the *B. graminis* RNAse effectors, the *P. graminis* AvrSr35‐like effectors, and the *Ustilago maydis* Tin2‐like effectors. Such study of these extremely expanded SUSS clusters provides insight in their evolutionary origin and the evolution‐driving processes as well as their putative function based on conserved motives and domain folds (Seong & Krasileva, 2023).

Contrasting apoplastic inhibitors, several effectors interfere with cytosolic host targets including NLRs. In solanaceous species, multiple members of the NRC‐helper network are inhibited by a secreted SPRY‐domain containing effector SS15 of the nematode *Globodera rostochiensis*, which inhibits the NRC resistosome assembly. Interestingly, the helpers NRC2, NRC3 and the NRC1 are sensitive to SS15 immune suppression, while NRC4 naturally evades its action. Studies in NRC2 showed that the NB‐ARC defines the NRC's SS15‐sensitivity. The NRC1^NB‐ARC^‐SS15 complex structure (PDB 8BV0) showed that SS15 binds a loop region within the NOD module of NRC1, which blocks its conformational activation. Sequence comparisons and mutagenesis revealed that the SS15 insensitivity of *N. benthamiana* NRC4 is mediated by only two polymorphic residues (P316 and K317) (Contreras et al., 2023). Also, some solanaceous NRC1 and NRC3 have evolved to evade SS15 immune suppression, based on their polymorphic residue 316 or corresponding (Contreras et al., 2023; Sugihara et al., 2025). As the ancestral NRC3 clade was SS15 sensitive and had evolved toward the K316 polymorphisms, this model demonstrated a new mode of gene‐for‐gene interaction where a plant effector target evades an effector suppression, expanding the widely accepted model where pathogen effectors evade the plant immune recognition (Flor, 1971). Therefore, the strategy to restore inhibited or overcome resistances builds the basis for creating durable resistances based on editing using new genomic techniques (NGT's) (Figure 1), which is pursued by start‐up companies like for example, Resurrect Bio.

## LEGAL CONSIDERATIONS IN PATHOGEN RESISTANCE ENGINEERING

The field cultivation, distribution, and consumption of genetically modified crops are still regulated by guidelines, limiting the utilization of bioengineered resistances in the field especially in the European Union (EU) and the United Kingdom (UK). By legislation, no distinction is made between genome‐edited plants using precise NGT's and transgenic plants in the EU, classifying both as genetically modified organisms (GMO's) requiring strict risk assessment of the European Food Safety Authority (EFSA) and banning them from cultivation and human consumption. Since 2023, simplified regulations of NGT's are under debate as the European Commission proposed new rules for the approval of plants harboring edits resembling natural alleles or which could be produced by traditional breeding techniques. A similar reform is expected in the UK, where GMO‐regulations for NGT plants is formally invalidated since 2023. Other regions including the United States of America (USA) and India already have simplified regulations to approve NGT plants (e.g., SECURE). Transgenic plants are still strictly regulated in the EU and the UK, but regulations are more flexible in the USA, China, India, and Brazil where transgenic plants are not banned for cultivation and distribution, only requiring proper labelling by a case‐for‐case risk assessment (Figure 1) (information taken from: Global Gene Editing Regulation Tracker and Index; 01.09.2025; https://crispr‐gene‐editing‐regs‐tracker.geneticliteracyproject.org/).

## ENGINEERING OF ROOT SYMBIOSIS

**Figure 2:**
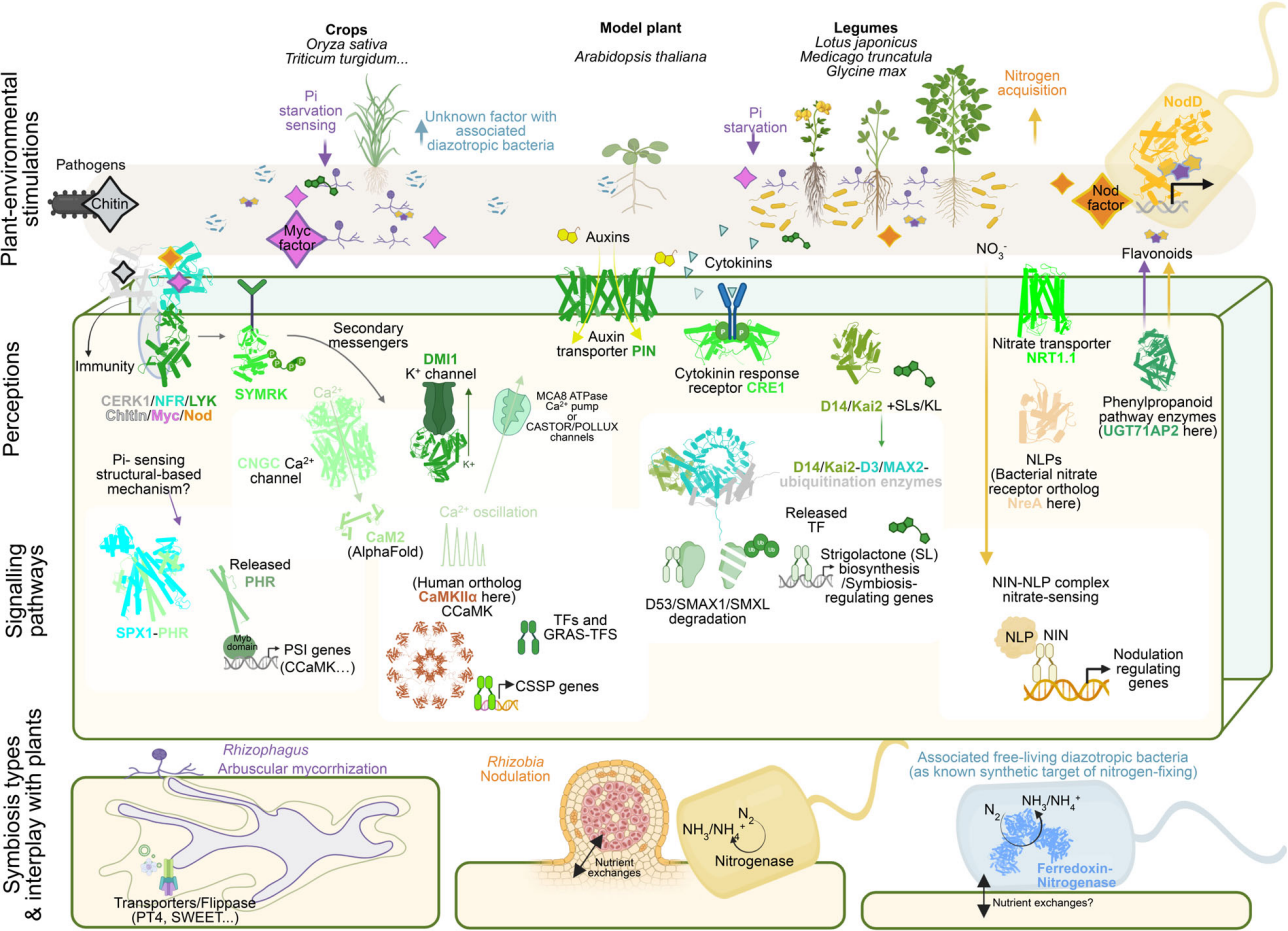
Structure zoo of the plant–microbe symbioses. Orchestrations of four perspectives describing in this review are shown as plant–environmental stimulations, perceptions of plant–microbe signals, signaling pathways for symbiotic regulation, and common symbiotic types with interplays between host plants. All the representative structures are listed in the Table 2. Represented protein structures were taken from the RCSB Protein Data Bank while NodD and CaM2 were from AlphaFold database. The visualization was using Chimera Version 1.18. Illustrations were taken from BioRender.

**Figure 3:**
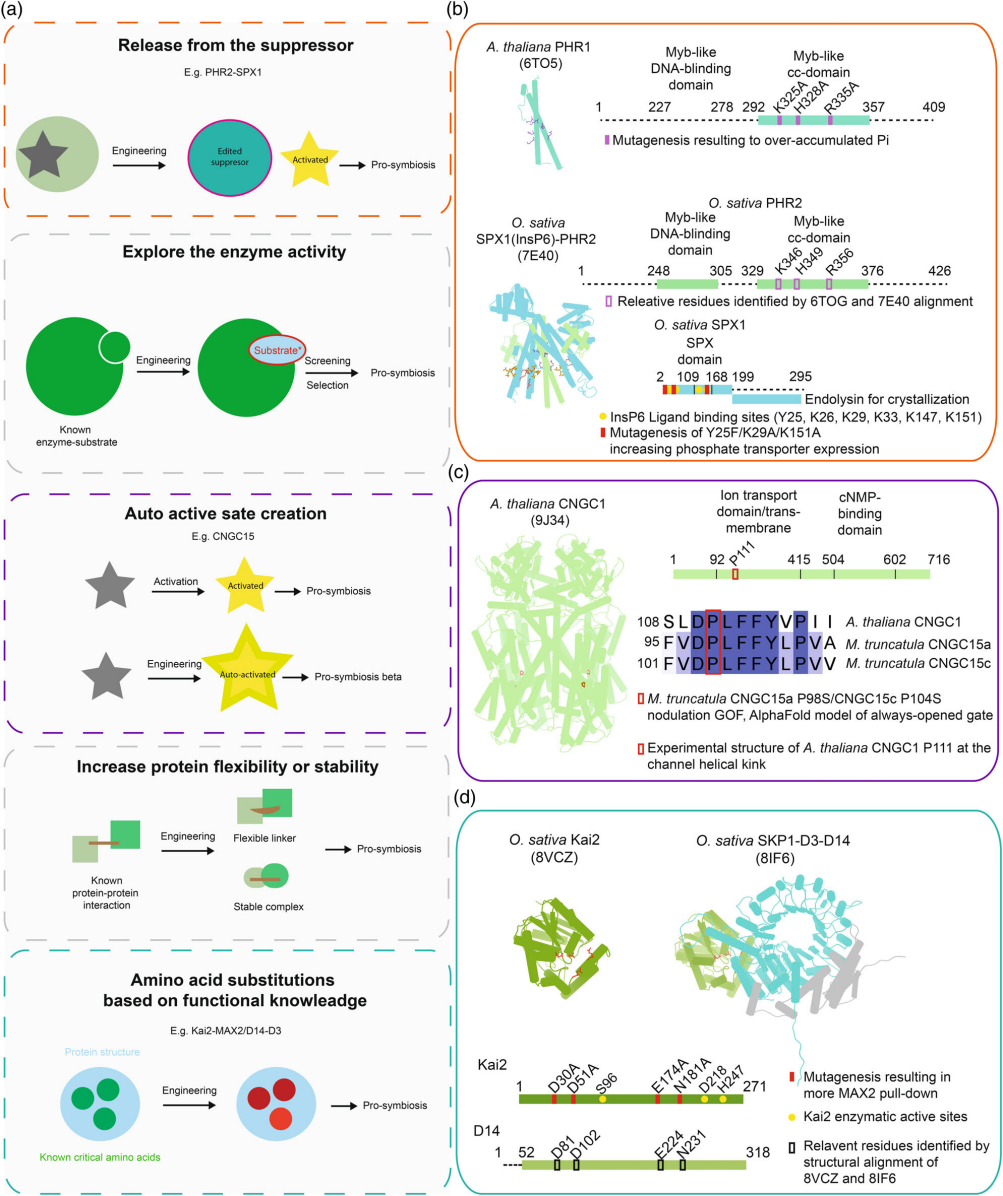
Strategies and selected examples for structural‐based engineering to promote plant–microbe symbioses. (a) Five strategies are shown here. (b), (c), and (d) are the examples of strategy ‘release from the suppressor’, ‘auto active state creation’, and ‘amino acid substitutions based on functional knowledge’ respectively. The domains, the structures, and the mutational information are present, based on the selected references discussed in this review. Represented protein structures were taken from the RCSB Protein Data Bank (RCSB.org). The visualization was using Chimera Version 1.18.

In contrast to the plant–pathogen interaction topic, where many practical engineering applications already exist, the plant symbiotic topic is expected to gradually attract increasing attention on structural design. Arbuscular mycorrhization symbioses (AMS) and root nodulation symbioses (RNS) are two major types of plant–microbe root symbiosis that have been studied. Symbiosis establishment can be viewed in two phases: (1) a pre‐symbiotic phase, where environment‐ and genotype‐specific signals of host and symbiont determine the initiation of the interaction, and (2) a committed phase, where sustained signal‐ and resource exchange supports the partnership. The following section discusses both stages and highlights how protein structural insights (Figure 2; Table 2) can guide trait engineering (Figure 3) in crops for improved symbiosis in agriculture.

**Table 2:** Structural studies of proteins involved in plant–symbiont interactions and the relative orthologs from other species

Plants/Microbes/Human	Protein(s)	PDB	References	Method	Comments
*A. thaliana*	PHR1	6TO5	Ried et al. (2021)	X‐ray	
*O. sativa*	SPX‐PHR2	7E40	Zhou et al. (2021)	X‐ray	With InsP6
*S. carnosus*	NreA	4IUK	Niemann et al. (2014)	X‐ray	Structurally compared with plant NIN in Liu, Liu, et al. (2022)
*E. coli*	NarX	3EZH	Cheung and Hendrickson (2009)	X‐ray	
*Synechocystis* sp.	NrtA	2G29	Koropatkin et al. (2006)	X‐ray	
*A. thaliana*	NRT1.1	5A2N	Parker and Newstead (2014)	X‐ray	
*S. baicalensis*	UGT71AP2	8HOJ	Wang et al. (2024)	X‐ray	
*A. thaliana*	CERK6	5LS2	Bozsoki et al. (2017)	X‐ray	
*L. japonicus*	LYK3	6XWE	Bozsoki et al. (2020)	X‐ray	
*M. truncatula*	NFR5	7AU7	Gysel et al. (2021)	X‐ray	LyM1‐3 domains
*O. sativa*	CERK1	7VS7	Xu et al. (2023)	X‐ray	With chitin
*L. japonicus*	NFR1	8S79	Hansen et al. (2024)	X‐ray	Pseudokinase
*M. truncatula*	LYR4	8PS7	Simonsen et al. (2025)	X‐ray	Pseudokinase
*L. japonicus*	SYMRK	8PEH	Abel et al. (2024)	X‐ray	Kinase domain
*M. truncatula*	DMI1	7VM8	Liu, Lin, et al. (2022)	X‐ray	Soluble C‐terminus
*A. thaliana*	CNGC1	9J34	Wang et al. (2025)	EM	
*M. truncatula*	CNGC15b	9KCU	Xu et al. (2025)	EM	
*H. sapiens*	CaMKIIα	5U6Y	Myers et al. (2017)	EM	Ortholog of plant CCaMK
		8T15	Chien et al. (2024)	EM	
*A. vinelandii*	MoFe‐Nitrogenase	9CJB‐9CJF	Warmack and Rees (2024)	EM	Alkaline turnover steps
*R. capsulatus*	FeS‐Nitrogenase	8OIE, 8PBB	Schmidt et al. (2024)	EM	−/+ catalytic components
*A. vinelandii*	MoFe‐nitrogenase	9CTZ 9CU0‐9CU2	Narehood et al. (2025)	EM	−/+ FeS/Ferredoxin
*R. tropici*	MprF	6LVF, 7DUW, 6LV0	Song et al. (2021)	EM X‐ray EM	With amino‐acid‐lipids (LysPGs)
*C. elegans*	BLTP	9CAP	Kang et al. (2025)	EM	Lipid‐transport bridge
*S. cerevisiae*	ScPho90	8R33‐35	Schneider et al. (2024)	EM	Phosphate transporter −/+ substrates
*A. thaliana*	SWEET13	5XPD	Han et al. (2017)	X‐ray	Structurally compared in Anjali et al. (2020).
*O. sativa*	SWEET2b	5CTG	Tao et al. (2015)	X‐ray	
*A. thaliana*	PIN1	7Y9T‐V	Yang et al. (2022)	EM	Apo, NPA‐/IAA‐bound
	PIN8	7QP9/A/C	Ung et al. (2022)	EM	apo, IAA‐/NAA‐bound
*A. thaliana*	CRE1	7P8C	Tran et al. (2021)	X‐ray	Receiver domain
*M. truncatula*		7P8D, 7P8E			
*O. sativa*	D14‐D3‐D53‐SKP1	8IF6	Liu et al. (2023)	EM	With *rac*‐GR24
*A. thaliana + O. sativa*	D14‐D3‐ASK1	5HZG	Yao et al. (2016)	X‐ray	Chimera
*A. thaliana + O. sativa*	SKP1‐MAX2	7SA1	Tal et al. (2022)	X‐ray	Chimera
*O. sativa*	KAI2	8VCZ, 8VD1	Guercio et al. (2024)	X‐ray	Apo, with MPD
*A. thaliana*	D14	8VD3			Apo form
*O. sativa*	D14	4IH9	Zhao et al. (2013)	X‐ray	Apo form
		9JQG	Zhang, Wang, et al. (2025)	X‐ray	With cyclo‐Leu‐Pro
		5DJ5	Zhao et al. (2015)	X‐ray	With *rac*‐GR24

The species names listed sequentially here are *Arabidopsis thaliana*, *Staphylococcus carnosus*, *Escherichia coli*, *Synechocystis* sp., *Scutellaria baicalensis*, *Lotus japonicus*, *Medicago truncatula*, *Homo sapiens*, *Azotobacter vinelandii*, *Rhodobacter capsulatus*, *Rhizobium tropici*, *Caenorhabditis elegans*, *Saccharomyces cerevisiae*, and *Oryza sativa*. Methodologies to determine the structures listed here are X‐ray (X‐ray crystallography) and EM (cryogenic electronic microscopy). The colour usage in table is for distinguishing plants, microbes, and human.

Plants have evolved intricate uptake mechanisms for macro‐ and micronutrients to spur their growth (Moreno Jimenez et al., 2024). Under nutrient limiting conditions, competent plants inform and attract symbiotic microbes from their surroundings to specifically overcome a certain nutrient limitation. Both, the plant hosts and the co‐evolved specific beneficial microbes possess receptors and release certain infochemicals including hormones, peptides, carbohydrates, or proteins to communicate to each other ‘readiness’ for symbiosis establishment.

## PHOSPHATE STARVATION AND ARBUSCULAR MYCORRHIZA

Phosphorous is an important macronutrient for plants and is taken up by the root as inorganic phosphate (Pi). Members of the conserved MYB‐coiled‐coil (MYB‐CC) transcription factor family phosphate starvation response (PHR) are established through genetic experiments as master regulators of phosphate homeostasis in *O. sativa*, *A*. *thaliana*, and several other species (Das et al., 2022; Ren et al., 2025; Ried et al., 2021).

Under Pi starvations, plants attract the arbuscular mycorrhizal fungus (AMF) *Rhizophagus irregularis* to promote Pi uptake. In the common model for AMS, *Lotus japonicus* (*L. japonicus*), PHR2 regulates strigolactone biosynthesis, the common symbiosis pathway, and nutrient exchange. Transcriptomic profiling showed that AM‐related phosphate starvation‐induced (*PSI*) gene expression differs between wild‐type and *phr2* plants at low Pi, but these differences disappear in *PHR2*‐overexpressing plants under high Pi conditions (Das et al., 2022). These results underscore the importance of fine‐tuning Pi homeostasis when engineering crops for improved symbiosis.

Structure‐informed engineering toward phosphate starvation response could come from knowledge of the PHR molecular mechanism. In its resting state at low and physiological Pi conditions, PHR2 forms homodimers through CC domain bundling and acts as a transcriptional activator of *PSI* genes through DNA‐binding of its MYB domain (Ried et al., 2021; Rubio et al., 2001; Zhou et al., 2021; Zulfiqar et al., 2025). Similarly, the MYB domain was also shown to be essential for DNA‐binding of PHR1 from *A. thaliana* (PDB 6TO5) (Jiang et al., 2019). At elevated Pi concentration, PHR homodimers are disrupted and scavenged from their DNA‐binding sites by transcriptional repressors with an SYG1/Pho81/XPR1 (SPX) domain (Das et al., 2022). Structure of *O. sativa* PHR2 in complex with the SPX1 revealed that the Pi‐level linked molecules, inositol pyrophosphate (InsP6), bind at the proteins' interface, forming an InsP6‐SPX1‐PHR2‐ternary complex (PDB 7E40) (Zhou et al., 2021).

Future engineering of PHR2 function should proceed cautiously, as disruption of PHR2 dimers can trigger severe Pi mis‐regulation. Promisingly, gene expression of the *O. sativa Phosphate Transporter 2* (*PT2*) significantly increased by introducing mutations in the SPX1 InsP6‐binding site (Y25F/K29A/K151A) in the mutant *spx1 spx2* background (Zhou et al., 2021). Given that members of the phosphate transporter (PT) family show variable response to AMF (Paszkowski et al., 2002), systematic testing of PT expression under engineered PHR2/SPX1 variants would be valuable. Furthermore, recent findings that *M. truncatula* SPX1 and SPX3 regulate AM symbiosis independently of PHR2 (Wang et al., 2024) highlight that species‐specific regulatory circuits must be considered.

## NITROGEN ACQUISITION AND NODULATION

Nitrogen is essential for plant growth, and some species have evolved specialized symbioses with nitrogen‐fixing bacteria to supplement their nitrogen needs. This interaction is most advanced in root nodule‐forming plants and most extensively studied in the model legumes *L. japonicus*, *M. truncatula*, and in *G. max*. Similar to phosphate signaling, nitrogen availability tightly regulates symbiotic outcomes: low external nitrogen promotes, whereas high nitrogen suppresses association with rhizobia in legumes (van Noorden et al., 2016). Among the nitrogen regulatory pathways, nitrate (${\mathrm{NO}}_{3}^{-}$) signaling is mostly characterized. In legumes, the transcriptional master regulator Nodule Inception (NIN) interacts with NIN‐like proteins (NLPs) in the nucleus in response to nitrate (Lin et al., 2018). This NIN‐NLP complex represses root nodule marker genes and their loss‐of‐functional mutation leads to nitrate‐resistant nodulation (Schenk et al., 2020). Although the precise molecular mechanism of nitrate sensing by the NIN‐NLP complex remains unresolved, structural insights come from homologous bacterial and cyanobacterial proteins known to bind nitrate, including NreA from *Staphylococcus carnosus*, NarX from *Escherichia coli*, and NrtA from *Synechocystis* sp. (PDB 4IUK, 3EZH, 2G29) (Liu et al., 2022).

Beyond transcriptional control, nitrate perception also depends on membrane‐localized transporters of the NRT1 low‐affinity and NRT2 high‐affinity families. The best‐characterized example is the major facilitator superfamily (MFS) transporter *A. thaliana* NRT1.1 (PDB 5A2N) (Parker & Newstead, 2014). NRT1.1 can shift between low‐ and high‐affinity states through phosphorylation of amino acid T101, providing plants with a rapid means to adapt to fluctuating nitrate availability. A previous preprint in *A. thaliana*, as a non‐legume plant, strikingly presents rhizobia attaching to root surfaces leading to root morphological changes dependent on the nitrate signaling pathway with NLPs and NRT1.1 involved (Schenk et al., 2020). Moreover, the legume NRT1 and NRT2 families are expanded (Pellizzaro et al., 2017), and several members are upregulated during nodule development in *L. japonicus* (Vittozzi et al., 2021) and *M. truncatula* (Luo et al., 2023; Pellizzaro et al., 2015). Notably, *M. truncatula* NRT2.1 is required for nitrate‐dependent inhibition of nodulation, underscoring a regulatory role for NRT transporters in legume–rhizobia symbiosis.

Collectively, structure‐guided engineering of nitrogen responses, particularly nitrogen‐resistant nodulation in legumes, are potential avenues toward symbiosis engineering for agriculture.

## RHIZOBIA DETECT HOST‐DERIVED SIGNALS

To recruit nitrogen‐fixing bacteria, legumes release flavonoids into the rhizosphere. These secondary metabolites that are derived from the phenylpropanoid pathway and frequently exist in their aglycon and their glycosylated form that have different biological activities (Shen et al., 2022). Flavonoids, and certain glycoside‐flavonoids, were shown to act as determinants of host specificity and presumably dictate genotype compatibility between rhizobial strains and plant hosts (Spaink et al., 1987). Yet, a complete functional characterization and determination of flavonoid‐rhizobia specificity is lacking due to the limited characterization for flavonoid production in the host plant. On the other hand, perception of specific flavonoids initiates a cascade of rhizobial responses through the activation of the flavonoid receptor and transcription factor NodD, which in turn induces the expression of genes required for Nod factors biosynthesis, effector secretion, and type III pili formation. Next to the recruitment of rhizobia, flavonoids also have roles in root auxin transport, inhibition of competitive rhizosphere microbes, promotion of nodule development, and number (Dong & Song, 2020).

Structure of the plant UDP‐glycosyltransferase UGT71AP2 from *Scutellaria baicalensis* revealed a dynamic active site formed by two dynamic Rossmann‐like domains, enabling structure‐guided enzyme engineering. By introducing carboxyl or hydroxyl groups at residues to stabilize the flavanone backbone, Wang et al. created mutants with altered regio‐selectivity. The triple amino acid substitution L138T/V179D/M180T achieved 92% 2′‐*O*‐glycosylation compared with 46.2% for the wild‐type enzyme, demonstrating how rational design can redirect catalysis for future engineering applications (PDB 8HOJ) (Wang et al., 2024).

Compared with slow and complex plant breeding, engineering symbionts like rhizobia offers an alternative. In *Rhizobium leguminosarum* bv. *viceae*, a stop‐codon mutation at residue R98 truncated the repressor domain of NodD, yielding a flavonoid‐independent, constitutively active NodD that successfully produced nodules with leghemoglobin and nitrogenase activity, markers of productive nodule formation (Haskett et al., 2025) (NodD model is available with Uniprot P04681 on AlphaFold database).

## PLANT RECEPTORS DETECT SYMBIONT‐DERIVED SIGNALS

To distinguish symbionts from pathogens, plants use a diverse but overlapping set of receptors, with the specific combination of activated receptors determining the outcome. Famous examples for such receptors are the Lysine motif (LysM) receptors, including Chitin Elicitor Receptor Kinase 1 (CERK1) and Symbiosis Receptor‐like Kinase (SYMRK). For instance, pathogenic microbes release chitin oligomers, while symbionts such as AM fungus and rhizobia release lipochitooligosaccharides (LCOs, also termed Myc and Nod factors). Both types of molecules are detected by receptors with three extracellular LysM1‐3. Whereas chitin is recognized by the plant immunity LysM receptor CERK, LCOs are bound by NFR receptors (also known as LYKs or LYRs). These LCO‐recognizing receptors have been extensively studied functionally through domain swapping, crystallographically, biochemically, and phenotypically in context of symbiont inoculation (Bozsoki et al., 2020; Gysel et al., 2021; Hansen et al., 2024; Simonsen et al., 2025).

Structures of *A. thaliana* CERK6 (Bozsoki et al., 2017) (PDB 5LS2) and *L. japonicus* LYK3 (PDB 6XWE) guided structure‐based domain swapping of CERK6 and NFR1 in *L. japonicus*. These domain swaps showed that the CERK6 and NFR1 extracellular LysM1 domain is the critical position to distinguish LCO and chitin ligands in these receptors (Bozsoki et al., 2020). In addition, structure of the *M. truncatula* NFR5 LysM1‐3 ectodomains was solved (PDB 7AU7) and showed that a hydrophobic patch in the LysM2 domain also determines specificity in this LysM receptor. Interestingly, NFR5 overexpression in *M. truncatula* carrying substitutions at L147D/L154D rescues nodulation in the *nfp* mutant highlighting the role of the LysM2 hydrophobic patch. Consequently, the hydrophobic patch in LysM2 is absent from other pathogen‐related and chitin‐binding LysM receptors. Comparison of binding kinetics of *L. japonicus* LYS11 and *M. truncatula* NFR5, with their respective rhizobium‐derived LCO ligands, and CERK with its pathogen‐derived chitin ligand, showed striking differences and increased on/off rates for LCO receptors suggesting their proof‐reading function (Gysel et al., 2021). Interestingly in *O. sativa*, chitin and LCO perception are respectively mediated by CERK1 directly (Carotenuto et al., 2017) and together with Myc factor receptor 1 (He et al., 2019). Comparison to natural alleles and the structure of CERK1 with chitin–hexamer ligand (PDB 7VS7) was used to generate a double amino acid substitution in the LysM2 domain (I118T/S121T) to increase affinity against to chitin ligands (Xu et al., 2023). Such data will be instrumental in efforts to engineer improved mycorrhiza establishment without compromising plant resistance in monocot crops.

CERKs carry active kinase domains, which signal through co‐receptor LYK‐induced autophosphorylation (Fukuda et al., 2024; Mittendorf et al., 2024). In *O. sativa*, such a LYK co‐receptor is named chitin elicitor‐binding protein (CEBiP) (Shimizu et al., 2010). Similar to the LYK5 co‐receptor to CERKs, the *L. japonicus* LCO receptors NRF5 and NFR1 carry a pseudokinase domain with no apparent enzymatic activity (Wong et al., 2019). This was confirmed through structure‐guided mutational studies on *M. truncatula* LYR4 showing that a ATP‐binding mutant (A377F) and a kinase activity mutant (L392D) displayed clear response upon chitin octamers treatment (PDB 8PS7) (Simonsen et al., 2025). *L. japonicus* NRF5 and NFR1 possess a juxtamembrane motif mediating dimerization of these receptors (PDB 8S79) (Hansen et al., 2024). This assembly was later confirmed via nanobody approach then further guiding the discovery of the receptors pair in evolutionally distant *H. vulgare*, RLK4 and RLK10, which rescued the nodule organogenesis in *L. japonicus nrf1 nfr5* background (Rubsam et al., 2023).

SYMRK mutation was first isolated as a null allele for rhizobia colonization and later also shown to be a null allele for AMS (Endre et al., 2002; Gherbi et al., 2008; Stracke et al., 2002). This is due to its function as a co‐receptor with active intracellular kinase domain of *L. japonicus* NFR5 and NFR1 (Abel et al., 2024) (*L. japonicus* SYMRK PDB 8PEH). During activation, the SYMRK ectodomain appears to be auto‐cleaved (Antolin‐Llovera et al., 2014) but the exact role of this property of SYMRK remains ambiguous. Nevertheless, due to its central position in symbiosis signaling, SYMRK presents an attractive target for symbiosis trait engineering, even in cereal crops such as *O. sativa* (Li et al., 2018; Miyata et al., 2023) and *Zea mays* (Zhou et al., 2025). Furthermore, critical symbiotic roles of SYMRK phosphorylation direct downstream Early Phosphorylated Protein 1 (EPP1) have been recently investigated in AMS and RNS (Nørgaard et al., 2025; Rich et al., 2025). Furthermore, the engineered plants targeting on the SYMRK‐EPP1 interface showed spontaneous nodules (Nørgaard et al., 2025).

## SWITCH IT ON: THE COMMON SYMBIOSIS SIGNALING PATHWAY

Downstream of cell surface perception of symbionts is the so‐called common symbiosis signaling pathway (CSSP). The CSSP relies on nuclear calcium as a secondary messenger to inform from the plasma membrane to the nucleus. Perinuclear Ca^2+^ oscillations are generated through the coordination of several ion channels in perinuclear membranes. In *M. truncatula*, the sarco/endoplasmic reticulum Ca^2+^‐ATPase MCA8 works together with the Ca^2+^‐channels Cyclic Nucleotide‐gated Channel 15 isoforms (CNGC15a‐c; known as CASTOR in *L. japonicus*) and the non‐selective cation channel Doesn't Make Infections 1 (DMI1; known as POLLUX in *L. japonicus*) (Capoen et al., 2011; Charpentier et al., 2008, 2016). Together, these proteins drive the influx and efflux of Ca^2+^ generating rhythmic nuclear Ca^2+^ oscillations. These oscillations act as signals, activating downstream transcriptional responses for promoting symbiosis.

Recent structural studies have revealed how specific GOF mutations in symbiotic Ca^2+^ channels modulated signaling. Soluble C‐terminal DMI1 structure offers an explanation for the spontaneous nodule phenotype of the S760N GOF mutant, showing that the mutation likely stabilizes specific salt bridges between DMI1 subdomains, locking the channel in the active state (PDB 7VM8) (Liu, Lin, et al., 2022). Similarly, a P‐to‐S substitution in CNGC15 produced a GOF variant that autonomously triggers Ca^2+^ oscillations even without DMI1. AlphaFold2 modeling suggested that this mutation (P98S and P104S in CNGC15a and c, respectively) disrupts a kink in the S1 transmembrane helix, causing a conformational rearrangement that keeps the channel open (Cook et al., 2025). Complementary structures of *A. thaliana* CNGC1 (PDB 9J34) (Wang et al., 2025) and *M. truncatula* CNGC15b (PDB 9KCU) (Xu et al., 2025) further illuminate cation selectivity and gating mechanisms. Together, these insights highlight how single amino acid mutations can reprogram channel activity, offering a framework for engineering symbiotic efficiency.

Among the CNGC binding partners, the calcium‐sensing protein calmodulin (CaM) was revealed to regulate the local channel calcium‐dependent feedback through CNGC (Dietrich et al., 2020). In *M. truncatula*, the CNGC15 interacts with CaM2 in the presence of calcium. Functional studies *in planta* of the designed CaM2 R91A strikingly enhanced the nodulation but not AM colonization, explained by an increased CaM2 flexibility, further accelerating the nuclear calcium oscillation frequency (Del Cerro et al., 2022) (CaM2 model is available with gene assessment Medtr5g088320 on AlphaFold database).

Calcium/Calmodulin‐dependent protein kinase (CCaMK), the activated consequence of nuclear calcium oscillation, also interacting with CaM, plays critical roles in plant symbiosis (Yuan et al., 2022). Although the activation mechanism and atomic structure of plant CCaMK remain elusive, future engineering design could consider analogy with the human calcium/calmodulin‐dependent protein kinase II isoform α (CAMKIIa) (PDB 8T15; 5U6Y) (Chien et al., 2024; Myers et al., 2017). CCaMK phosphorylates the transcriptional factor CYCLOPS in the presence of DELLA to activate the transcription of symbiotic‐related genes, such as Reduced Arbuscular Mycorrhiza 1 (RAM1) (Pimprikar et al., 2016), CTTC Motif‐binding Transcription Factor 1 (CBX1) and WRINKLED1‐like families WRI5 a‐c in an AM manner (Paries et al., 2025), while NIN (Singh et al., 2014) and ERN1 encoding AP2/ERF transcription factor (Cerri et al., 2017) in the nodulation manner, and the Calcium Binding protein 1 (CBP1) in both manners (Gong et al., 2022).

GRAS proteins are plant‐specific transcriptional factors governing plant development that are named from the abbreviations of gibberellic acid‐insensitive, repressor of GAI, and SCARECROW (Hirsch & Oldroyd, 2009). GRAS proteins form constitutive homo/heterodimers, each monomer containing N‐terminal variable disordered regions, and the C‐terminal GRAS domain forms an α‐helical cap and an α/β core subdomain (Hakoshima, 2018; Jaiswal et al., 2022). The recent ROSETTafold model of one GRAS protein, RAM1, in interaction with CBX1 and WRI5b provides information for future design of the binding interface, which was validated through interface mutagenesis, particularly in decreasing AM colonization (Paries et al., 2025).

## LIVING WITH EACH OTHER: EXCHANGE OF RESOURCES AND SIGNALS BETWEEN SYMBIOTIC PARTNERS

Plants lack functional nitrogenase to convert atmospheric dinitrogen into bioactive ammonia, so plants rely on rhizomicrobes for nitrogen. Thus, engineering the nitrogen‐fixing nitrogenase machinery of rhizosphere free‐living diazotrophic bacteria represents a state‐of‐the‐art approach in the synthetic nitrogen fixation (Bennett et al., 2023). The resurrection evolution via the ancient enzyme replacements with modern free‐living diazotrophic *Azotobacter vinelandii* nitrogenase machinery (Garcia et al., 2023), the structural time‐course reactional turnover of the *A. vinelandii* nitrogenase components (PDB 9CJB‐9CJF) (Warmack & Rees, 2024), and the structure of phototrophic‐linked nitrogen‐fixing nitrogenase from purple bacteria *Rhodobacter capsulatus* (PDB 8OIE, 8PBB) (Schmidt et al., 2024) provide us the critical cores for future engineering. Nitrogenase reactions are dependent on the oxygen‐sensitive metalloclusters. These clusters are always coordinated by conserved residues C and H, which shall avoid changes in future engineering (Schmidt et al., 2024; Warmack & Rees, 2024). Electrons from ferredoxins transferred to di‐nitrogenase reductase and the oxygen protection provided by ferredoxin homodimers define the turnover rate of the entire system, which was evidenced by the newly published *A. vinelandii* ferredoxin‐nitrogenase filamented complexes (PDB 9CTZ, 9CU0‐2) (Narehood et al., 2025). In our opinion, the interfaces of each nitrogenase component and the ferredoxin are also untouchable for future designs.

Phospholipids are critical signals for root development (Oubohssaine et al., 2025). At the molecular level, phospholipids generally function upstream of nuclear Ca^2+^ oscillations (Xue et al., 2009), which are essential for the CSSP pathway. Recently, a transcriptomic study reported a highly regulated lipid metabolism during *Medicago sativa* L. nodulation (Lu et al., 2025). However, the source of such lipids and possible transportation route are still unclear. Based on the bacteria's wide distribution of multiple peptide resistance factor (MprF), research that drove our interest was focused on the synthetic mechanism of a certain amino acid‐conjugated type of lipid from a nitrogen‐fixing rhizobium, *Rhizobium tropici*. Structure of dimerized MprF consisted of the lipid synthase domains and lipid flippase domains. By mutating the bottleneck of the lipid flipping route, it accumulated more amino‐acid‐conjugated lipids, making this a candidate for engineering (PDB 6LVF, 7DUW, 6LV0) (Song et al., 2021). For transporting the hydrophobic lipids between cellular solvent environments, the lipid‐bridge is a unique molecular architecture composed of evolutionarily conserved bridge‐like lipid‐transport proteins (BLTPs) that might be a potential study target similar to MprF structural feature to understand how lipids travel between symbionts and plants, especially with the recent publication about the *Caenorhabditis elegans* BLTP LPD‐3 structure with lipids arraying in this tunnel‐like bridge (PDB 9CAP) (Kang et al., 2025). PHR and the phosphate starvation response also have negative effects on nodulation (Lu et al., 2020; Muller, 2021; Singh et al., 2025; Wang et al., 2020), suggesting that nodules play an indirect role in the uptake of soil Pi via an underestimated molecular mechanism involving PTs and purple acid phosphatase. Nevertheless, the underestimated connection between phospholipid or lipid metabolism, lipid flipping, and nodulation needs to be further clarified.

Mutualistic symbioses such as AMS rely on nutrient exchange between host and microbes, especially via transporters. AMS‐involved transporters have been summarized in the previous review (Anckaert et al., 2024), most of these transporters in crops have been considered as engineering targets not only because of their symbiotic functions but also for regulating plants' anti‐abiotic stress and anti‐pathogens (Yadav et al., 2021). The phosphate transporters PT4 from *L. japonicus* and *M. truncatula* are critical for arbuscule development (Harrison et al., 2002; Volpe et al., 2016), in contrast, PT6 involves phosphate uptakes in roots and nodules (Cao et al., 2020). Previous estimation suggested that PT4 belongs to the low‐affinity inorganic PT classification (Harrison et al., 2002), due to the current lack of structural information, we may borrow the knowledge from *Saccharomyces cerevisiae* low‐affinity PTs ScPho90 (PDB 8R33, 8R34, 8R35) (Schneider et al., 2024) for future engineering design. Sugar exporters from the SWEET transporter family functioning in symbiotic transcriptional reprogramming and arbuscule maintenance have been respectively reported in *S. lycopersicum* (Manck‐Gotzenberger & Requena, 2016) and *M. truncatula* (Harrison et al., 2002). Appreciating the broad functional spectrum of the plant SWEET family besides symbiosis, structural‐guided regulation on SWEET has been described in a previous review (PDB 5XPD, 5CTG) (Anjali et al., 2020) that we can mirror onto symbiotic‐related manipulation in the future.

## PLANT HORMONES INTERFERE WITH SYMBIOSIS SIGNALING AT MULTIPLE LEVELS

Plant hormones, such as auxins, cytokinins, gibberellins, and strigolactones (SLs), orchestrate various physiological phenomena in plants, including symbiosis (Boivin et al., 2016; Frugier et al., 2008; Miura et al., 2024; Nomura et al., 2024). Auxin transporters belong to the protein PIN‐FORMED (PIN) family that is classified under the Na^+^/H^−^ transporters superfamily. Symbiosis‐related phenotypes have been reported for mutants of *M. truncatula* PIN2‐4 and PIN10, and for *A. thaliana* PIN2 (Ng et al., 2015). Recent *A*. *thaliana* PIN1 and PIN8 structures have elucidated the auxin transport mechanism acting through conformational change of the scaffold domains. Two conserved P residues (PIN1 P111 and P579; PIN8 P116 and P3256), coordinating the benzene ring of auxins (PDB 7QP9, 7QPA, 7QPC; 7Y9T‐V) (Ung et al., 2022; Yang et al., 2022). Conclusively, the function of PINs in symbiosis makes them attractive candidates for structure‐guided tuning of auxin transport and root symbiosis.

In nodulation, cytokinin is recognized by the Cytokinin Response 1 (CRE1) receptor. CRE1 has four domains: an extracellular cyclases/histidine kinases associated sensory extracellular domain (CHASE domain), a transmembrane fragment, a histidine kinase (HK) domain, and receiver domain (REC). The CRE1 dimer accepts the cytokinin signals at the CHASE domain, then triggers the sequential autophosphorylation of its HK and REC domains, to initiate signal transduction. The determined structures of REC domain dimers (PDB 7P8C‐E) (Tran et al., 2021), respectively from *A. thaliana* and *M. truncatula*, illustrate that the intracellular REC domains of CRE1 are topologically intertwined, which could inform tunable nodulation via disruption of REC‐mediated CRE1 dimerization.

Upon Pi starvation, strigolactones (SLs) are released into the rhizosphere to stimulate the communication between AM fungi and host plants. Strigolactones are molecules contain tricyclic lactones (ABC rings) linked to a hydroxymethyl butanolide (D ring) as basic scaffolds recognized by the SL receptors, DWARF14 (D14) and D14‐like (D14L). Next to SLs, the smoke‐derived Karrikins are molecules based on D rings that are recognized by Karrikin Insensitive 2 (KAI2) receptor. The response to SLs and Karrikins is governed by the Ubiquitin‐Proteosome Pathway in a step‐wise process: (1) First, the SL and Karrikin receptors bind and hydrolyze their respective butanolide ligands inducing the interaction with DWARF3 (D3), which is a component of the E3 ubiquitin ligase complex formed by the proteins SKP1‐CUL1‐F‐box (SCF) or ASK1‐CUL1‐F‐box. D3 is also known as More Axillary Branches 2, MAX2. (2) This leads to the degradation of the suppressor of MAX2 (SMAX)‐like protein family that are negative regulators of transcription via the proteasome; including DWARF53 (D53) related to the D14 receptor, and SMAX1 related to the KAI2 receptor.  Strikingly, distinct conformations are captured in the three currently available structures of the SL‐related complexes, namely the D14‐D3‐D53‐SKP1 complex containing the synthesized SL‐like ligand *rac*‐GR24 (PDB 8IF6; Liu et al., 2023), the D14‐D3‐ASK1 complex (PDB 5HZG; Yao et al., 2016) and the D3‐MAX2 C‐terminal helix‐ASK1 complex (PDB 7SA1) (Tal et al., 2022). While switching the receptor specificity has turned out a successful strategy for structure‐based engineering of SL/KAR‐related signalling, the flexibility of the SCF complexes complicates attempts to modulate their function. Interestingly, MAX2 was pulled down more when the hydrophobicity of the *O. sativa* KAI2 binding pocket was increased by amino acid substitution (Guercio et al., 2024). Due to the structural conservation of D14 and KAI2 demonstrated by several crystal structures, namely D14 (PDB 4IH9) (Zhao et al., 2013) and KAI2 from *A. thaliana* and *O. sativa* (PDB 8VCZ, 8VD1, 8VD3) (Guercio et al., 2024), this promising design idea could be expanded to other SL/KAR receptors to modulate the SCF pathway intensity. Screening in *A. thaliana* for hypocotyl length as a KAI2‐dependent phenotype to identify novel KAI2‐binding compounds (Wang et al., 2025), might serve as a good example for future high‐throughput selection of receptor designs. A recent study (Zhang, Wang, et al., 2025) found that specific *O. sativa* root microbiota produced a compound with similarity to the D ring, namely cyclo‐Leu‐Pro, which activates SL signaling, leading to a longer primary root and increased tillering phenotype in rice through interaction with D14. Moreover, the mutagenesis based on structural comparison between the D14‐cyclo‐Leu‐Pro bound form (PDB 9JQG) and the previously determined *rac*‐GR24 bound form (PDB 5DJ5) (Zhao et al., 2015) provides a template for future protein engineering of D14, thereby allowing its specificity spectrum to be modified for both known ligands and novel synthetic ligands. Similar designs of KAI2 could pave the way for the application of symbiosis‐modulating compounds in an agricultural setting.

## CONCLUSION

Structural biology has exposed both the power and the limits of current plant–microbe engineering paradigms. While pathogen immunity research has demonstrated that receptor function, specificity, and regulation can be predictably redesigned, these successes also reveal that molecular recognition alone is insufficient: effective engineering must respect native signaling architecture, co‐adaptation, and physiological constraints. Symbiosis engineering now stands at an inflection point, where accumulating structural insight demands a shift from descriptive cataloging toward hypothesis‐driven design. A decisive opportunity arises from the rapid emergence of high‐quality pangenomes for major crop lineages, including legumes, Brassicaceae, cereals, and solanaceous species. These resources expose naturally evolved polymorphisms that encode species‐ and genotype‐specific competence for microbial interaction, offering an empirical design space shaped by selection rather than abstraction. Integrating pangenomic variation with structural models enables identification of permissive and restrictive interfaces, regulatory buffers, and evolutionary dead ends. Future advances will depend on coupling this evolutionary information with computational design and scalable functional screens to navigate trade‐offs between robustness, growth, and interaction specificity. In doing so, structure‐guided engineering may transition from isolated molecular feats to principled, evolution‐aware crop design.

## CONFLICT OF INTEREST

The authors declare no conflict of interest.

## Data Availability

Data sharing not applicable to this article as no datasets were generated or analysed during the current study.
